# Factors Involved on Tiger-Stripe Foliar Symptom Expression of Esca of Grapevine

**DOI:** 10.3390/plants10061041

**Published:** 2021-05-21

**Authors:** Francesco Calzarano, Giancarlo Pagnani, Michele Pisante, Mirella Bellocci, Giuseppe Cillo, Elisa Giorgia Metruccio, Stefano Di Marco

**Affiliations:** 1 Faculty of Bioscience and Technologies for Food, Agriculture and Environment, University of Teramo, Via Renato Balzarini, 1, 64100 Teramo, Italy; gpagnani@unite.it (G.P.); mpisante@unite.it (M.P.); 2 Veterinary Public Health Institute of Abruzzo and Molise Regions (IZSAM), Campo Boario, 64100 Teramo, Italy; m.bellocci@izs.it; 3 Department of Agronomy, Food, Natural Resources, Animal and Environment, University of Padova, 16-35020 Legnaro, Italy; giuseppe.cillo@studenti.unipd.it; 4 CNR, IBE, Via Gobetti 101, 40129 Bologna, Italy; elisa.metruccio@ibe.cnr.it (E.G.M.); stefano.dimarco@ibe.cnr.it (S.D.M.)

**Keywords:** grapevine, esca complex, wood disease control, leaf symptoms

## Abstract

Esca of grapevine causes yield losses correlated with incidence and severity symptom expression. Factors associated with leaf symptom mechanisms are yet to be fully clarified. Therefore, in 2019 and 2020, macro and microelement analyses and leaf reflectance measurements were carried out on leaves at different growth stages in a vineyard located in Abruzzo, central Italy. Surveys were carried out on leaves of both never leaf-symptomatic vines and different categories of diseased vine shoots. Never leaf-symptomatic and diseased vines were also treated with a fertilizer mixture that proved to be able to limit the symptom expression. Results showed that untreated asymptomatic diseased vines had high calcium contents for most of the vegetative season. On the contrary, treated asymptomatic diseased vines showed higher contents of calcium, magnesium, and sodium, at berries pea-sized, before the onset of symptoms. These vines had better physiological efficiency showing higher water index (WI), normalized difference vegetation index (NDVI), and green normalized difference vegetation index (GNDVI) values, compared to untreated asymptomatic vines, at fruit set. Results confirmed the strong response of the plant to symptom expression development and the possibility of limiting this response with calcium and magnesium applications carried out before the symptom onset.

## 1. Introduction

Esca of grapevine is a complex, destructive and widely spread disease. The disease includes the involvement of several microorganisms producing different types of wood deterioration at different age of the plant, from nursery to ageing vineyards. The disease has been commonly associated with tracheomycotic pathogens as *Phaeomoniella chlamydospora*, *Phaeoacremonium minimum* (or another species of *Phaeoacremonium*), and with the basidiomycete *Fomitiporia mediterranea,* or other species recently isolated in United States [1,2,3,4,5]. The complexity of the disease, especially for the role of pathogens, i.e., of *Botryosphaeriaceae* species [2], led to the proposal of a classification, although the fail to fulfill Koch’s postulates. In particular, Esca of grapevine was defined Esca complex and divided into five syndromes [6]. Three diseases, Brown wood streaking, Petri disease and grapevine leaf stripe disease (GLSD) were grouped as “grapevine phaeo-tracheomycosis complex”. White rot, the fourth disease of Esca complex, was mainly caused by *Fomitiporia mediterranea* [7]. The fifth disease, named as Esca proper, was considered the concomitant occurrence of tracheomycotic and white rot pathogens and relative wood alterations in the same plant, traditionally reported as “Esca of grapevine”.

Some studies where tiger-stripe symptom expression was associated with infections of tracheomycotic fungi, without the occurrence of white-rot necrosis, as for “young esca” in Australia, somehow supported this classification [8,9]. On the other hand, and more recently, a correlation between the amount of white-rot necrotic tissues and leaf symptoms was recorded in different studies [10,11,12,13].

The leaf blade of diseased plants showed light green chlorosis that can expand and coalesce in interveinal yellow red-brown stripes, displaying the so-called tiger-stripe symptoms [1,3]. In any case, incidence and severity of the foliar symptom expression demonstrated to be correlated with qualitative–quantitative yield losses [14,15]. Moreover, the symptoms occurrence and the consequent yield losses may vary from one season to another, regardless of the rate of woody tissue deterioration, and in relation to physiological, cultural, and environmental factors not completely cleared yet [9]. Meteorological factors can influence the occurrence of foliar symptoms in a given area and season as demonstrated for June and July rainfall, which appeared to be correlated to the foliar symptom expression [16,17]. Cultural factors also proved to play a role in foliar symptoms expression [18].

Studies hypothesized that foliar symptoms occurrence was based on the toxic metabolites produced by fungi in the wood [19,20,21]. Toxins can reach the canopy through the transpiration stream and contribute to induce leaf responses that leads to the formation of interveinal necrosis as the results of a hypersensitivity reaction [2,22,23]. However, further hypotheses on the occurrence of foliar symptoms were recently considered [24]. A functional disorder of sap flow, as sap disruption caused by the wood altered by pathogens [25], nongaseous embolisms or occlusions of vessels by gels and/or tyloses [26,27], and/or a role of annual infections on annual shoots [28] were postulated. These studies once again highlight the number and complex interaction of parameters associated with the outburst of foliar symptoms.

Phytoalexins, mainly present in symptomatic leaves at pre-bunch closure, were synthesized in symptomatic leaves as a consequence of the appearance of necrosis, and therefore did not seem effective in reducing symptom development [29]. This hypothesis seemed to be confirmed by the proportional increasing of phytoalexins with leaf symptoms increase [30]. Therefore, plants might react to the toxic metabolites with a kind of hypersensitivity reaction, in agreement with studies on the formation of anti-microbial compounds after the occurrence of symptoms [31].

The response of plant to the occurrence of symptoms seemed also associated with the nutritional status of the infected vines, and in particular with the calcium content [32,33]. Diseased vines treated with a fertilizer mixture based on calcium, magnesium, and seaweed significantly reduced foliar symptoms, synthesizing as well higher amounts of *trans*-resveratrol in the leaves earlier in the season, compared to untreated diseased vines. Therefore, an early increase of phytoalexins might contribute to the reduction of symptoms [34,35].

Precision agriculture is a site-specific informed management system that analyzes the factors that can vary over space and time in the production process, minimizing inputs (as water, fertilizers, plant protection products). The aim is to reduce the impact of agricultural practices on the environment, to increase the quality of products and the profitability of agriculture [36]. In this context, remote sensing techniques allow to detect physical or chemical characteristics of soil or plant organs either from proximal platforms (sensors) or remotely via satellite, drone, and aircraft [37].

Assessments are carried out in relation to the electromagnetic radiation emitted in a certain range of different wavelengths, called spectrum [38]. The bands of a spectrum in the optical domain make it possible to investigate phenomena connected with the photosynthetic capacity of crops. The optical domain [39] includes the visible, also called photosynthetically active radiation (PAR), with wavelengths between 400 and 700 nm, the infrared (IR) which in turn includes: near infrared (NIR, 700–1300 nm); short wave infrared (SWIR, 1300–2500 nm); midwave infrared (MWIR, 3000–8000 nm); thermal infrared (TIR, 7000–20,000 nm).

The most used vegetation indices to estimate the photosynthetic capacities of a crop based on the spectral response are the normalized difference vegetation index (NDVI), [40] and the green normalized difference vegetation index (GNDVI), [41]. These indices evaluated the leaf reflectance, expressed by the ratio between intensity of the reflected radiant flux and intensity of the incident radiant flux. NDVI considers the reflectance on wavelengths (λ) of the NIR (λ = 770 nm) and of the visible red (RED) (λ = 660 nm). GNDVI is calculated from the NDVI index by substituting the reflectance in the red with that in the green.

Through leaf reflectance measurements it is possible to detect different indices including the water index (WI), [42], as response of plant regarding specific infrared bands (R900/R970) so that water concentration in plants (PWC) and consequently the water stress can be estimated.

The aim of this study was to investigate parameters and mechanisms involved in complex processes of tiger-stripe symptoms formation, through leaf reflectance measurements and assessment of the main macro and microelements in leaves of both vines with different disease expressions and never leaf-symptomatic vines. The same analysis was carried out in diseased and never leaf-symptomatic vines treated with a fertilizer mixture capable of interfering with the foliar symptom expression, to obtain further information on the factors that regulate foliar symptoms expression.

## 2. Results

### 2.1. Leaf Fertilizer Applications and Foliar Symptom Surveys

**Figure 1:**
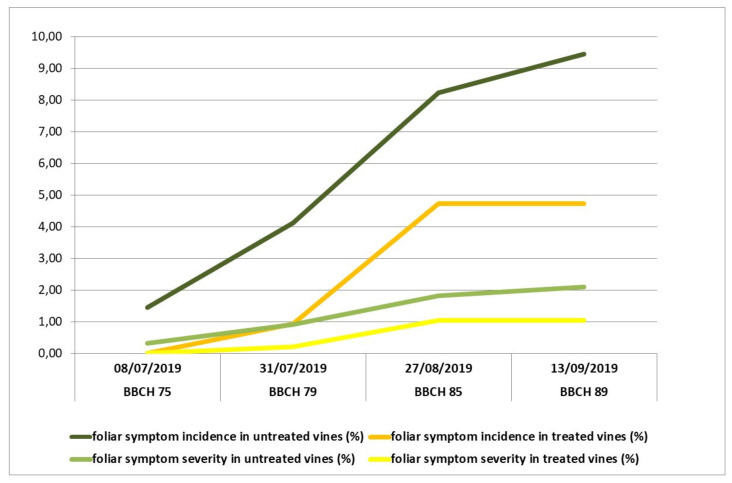
Incidence and severity of tiger-stripe foliar symptoms in vines treated with the fertilizer mixture and in untreated vines, in 2019, in Controguerra vineyard.

**Figure 2:**
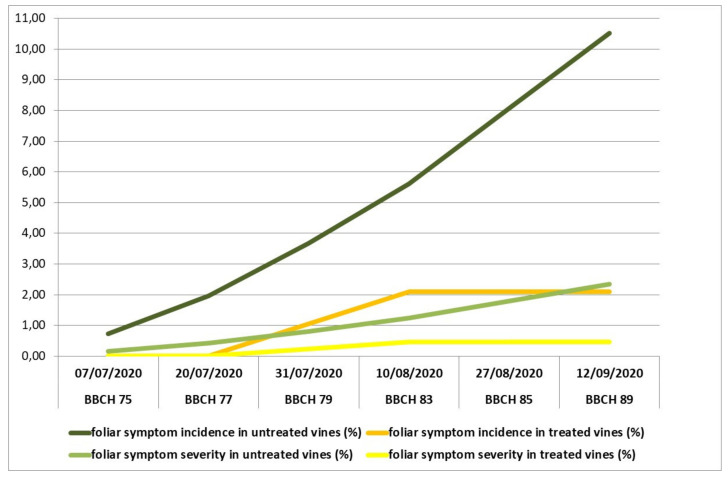
Incidence and severity of tiger-stripe foliar symptoms in vines treated with the fertilizer mixture and in untreated vines, in 2020, in Controguerra vineyard.

Both years of survey on incidence and severity of foliar symptoms in the Controguerra vineyard confirmed the dynamics of symptom expression, characterized by a remarkable increasing from the berries developing color stage. The effect of applications of the fertilizer mixture was noticeable in treated vines, in particular since the assessments carried out at majority of berries touching stage (31 July 2019 and 31 July 2020), and in the following growth stages, with the increasing of symptom expression in the vineyard (Figure 1 and Figure 2). Both in 2019 and 2020, the differences in symptoms of incidence and severity percentages between treated and untreated vines, evaluated at harvest (13 September 2019 and 12 September 2020), were statistically significant for both parameters, by means of Chi-square test for *p* = 0.05.

### 2.2. Leaf Reflectance Measurements

#### 2.2.1. Comparisons between Treated and Untreated Never Leaf-Symptomatic and Diseased Asymptomatic Vine Leaves

The results of reflectance measurements, carried out on leaves of both never leaf-symptomatic (NLS) and asymptomatic vines (AS), treated and untreated, showed higher values in leaves east facing and in full light (side A), compared to the leaves on side B, west facing and in shadow (Table 1 and Table 2).

Conversely, the trend of reflectance measurements carried out from growth stage 66 to 89, were similar in the two sides (A and B) in both years of survey (Table 1 and Table 2).

The NDVI and GNDVI values showed very similar trends and were always significantly lower in leaves of treated compared to untreated NLS and AS vines, in the first survey, at growth stage 66 (Table 1 and Table 2). On the contrary, in the third survey, at growth stage 71, the NDVI and GNDVI values of treated AS leaves appeared significantly higher than those of AS untreated leaves. In the same survey, the treated NLS leaves showed higher or similar values than those of untreated NLS leaves (Table 1 and Table 2). From the growth stage 75 to the last survey, growth stage 89, no differences were found between NLS and AS leaves. Few exceptions were observed only in 2020, at growth stage 77, for GNDVI (side A), and for NDVI (side B), with values of treated NLS and AS leaves significantly higher and lower than untreated NLS and AS leaves, respectively (Table 1 and Table 2).

In the first three surveys (growth stages 66, 69, and 71), values of the WI were higher in leaves of treated NLS and AS vines, compared to those of the corresponding untreated vines, both in side A and in side B. From the survey at growth stage 75 to the survey at growth stage 83 any difference between treated and untreated NLS and AS values was detected (Table 3). In growth stages 85 and 89 surveys, WI values were lower, in most of the cases significantly, in treated compared to untreated AS leaves (Table 3).

#### 2.2.2. Comparisons among Untreated Never Leaf-Symptomatic, Diseased Asymptomatic, and Diseased Symptomatic Vine Leaves

The leaf reflectance measurements carried out from the first leaf symptom appearance (8 July 2019 and 9 July 2020, growth stage 75) to the harvest (growth stage 89) on leaves of untreated never leaf-symptomatic (NLS) and asymptomatic (AS) vines, and on leaves of the different shoots of untreated symptomatic diseased vines (PRE-S, ASTIGR, and TIGR), did not show differences for both NDVI and GNDVI, except for significantly lower values in symptomatic leaves (TIGR) (Table 4).

In both years at growth stage 75 values of WI were significantly higher in AS leaves than in PRE-S, ASTIGR and TIGR leaves, but not different from values of NLS leaves. From surveys at growth stage 79 to the last surveys (growth stage 89), AS leaves showed higher values than the other leaves, in some cases significantly compared to those of PRE- S, ASTIGR, and TIGR (Table 5). 

### 2.3. Analysis of Macro and Microelements in the Leaf

#### 2.3.1. Macroelements

In untreated vines, only some differences in P content, not confirmed in both years of the study, were observed between leaves of the different categories. Only at harvest (growth stage 89), the P content was significantly higher in AS leaves than in leaves of the other categories of untreated shoots (Table S1).

P contents did not differ between leaves of treated plants, both in 2019 and 2020, except for TIGR leaves which showed higher values at growth stage 89 (Table S1).

In the comparison between treated and untreated vines, P contents were always lower (often significantly) in the treated AS leaves compared to untreated AS ones. Furthermore, in both years, at growth stage 85, lower values of treated compared to untreated ASTIGR leaves were recorded, although significantly only in 2020 (Table S1).

Contents of K did not show remarkable differences among leaves of the different categories of shoots. Some sporadic differences, such as those observed in 2019, between untreated PRE-S and TIGR leaves and treated NLS leaves, at growth stage 75, were not confirmed the following year (Table S1).

In both years, in untreated vines, Ca and Mg contents were higher in leaves of AS vines compared to NLS ones, from growth stage 75 to growth stage 85. In these stages the differences between NLS and AS were always significant for Ca. At growth stage 71 and 89 no significant differences were observed between the two categories of leaves. In leaves of ASTIGR shoots, Ca and Mg contents were similar in most of the cases to those of AS leaves. At growth stage 75, leaves of PRE-S shoots always had Ca and Mg contents lower than those of the other untreated leaves, significantly compared to AS leaves. In the following growth stages, Ca and Mg contents of PRE-S leaves increased and generally were not different from those of the other untreated leaves. Higher contents of Ca were recorded in treated compared to untreated TIGR leaves. This difference was significant at growth stage 75 in both years (Table 6).

In treated vines, Ca content was higher in AS leaves than in NLS ones, from 71 to 75 growth stage, to a significant extent in 2019, at growth stage 75, when Mg contents were also significantly higher in the AS leaves. Conversely, at growth stage 85, for both elements and in each of the two years of sampling, higher contents were observed in NLS leaves compared to AS ones, although significantly only for Mg in 2020. At this growth stage, Ca and Mg contents in leaves of the other categories of treated shoots were not different to those of NLS and AS ones, except for higher contents in TIGR leaves. At growth stage 89, no difference in Ca and Mg contents was detected among the various categories of treated leaves, with the exception of higher Ca levels in TIGR and ASTIGR leaves compared to NLS and AS leaves, recorded in 2019 (Table 6).

In both years of sampling, Ca and Mg contents were significantly higher in treated compared to untreated AS leaves, at growth stages 71 and 75. In the following sampling, at growth stage 85, a clear inversion of these results was observed, since significantly higher contents of Ca and Mg were recorded in untreated compared to treated AS leaves. In 2019, at growth stage 89, higher Ca and Mg contents in untreated than treated AS leaves were still detected. Unlike AS vines, Ca and Mg contents in leaves of NLS treated vines were not different compared to untreated NLS ones at growth stages 85 and 89 (Table 6).

#### 2.3.2. Microelements

In untreated vines, in 2019, at growth stage 75, Na content was significantly higher in NLS leaves, compared to AS, PRE-S, ASTIGR, and TIGR ones. In these categories of diseased vine, Na contents increased during the growing season. Therefore, in the following growth stages, a progressive leveling of Na content in the different categories was observed, which no longer differed from each other, with the exception of TIGR leaves. In both years TIGR leaves showed generally higher Na content than that of the other categories. Except for TIGR leaves, in 2020, at growth stage 75, the leaves of the different categories did not differ in Na content. However, as in 2019, in the following growth stages 85 and 89, no differences were noticed among NLS, AS, PRE-S, and ASTIGR leaves (Table 7).

In treated vines, except for some sporadic cases, no differences were recorded among NLS, AS, PRE-S, and ASTIGR leaves. Only TIGR leaves showed high Na contents especially recorded at growth stages 89, in 2019. At growth stages 85 and 89, AS, PRE-S, and ASTIGR leaves of treated diseased vines never differed from each other in both years of survey (Table 7).

In 2019 and 2020, Na contents appeared significantly higher in treated compared to untreated AS leaves, at growth stages 71 and 75. On the contrary, in the following growth stages, higher Na contents were recorded in untreated AS leaves. In 2020, treated NLS leaves had significantly higher content of Na at growth stages 71, 75, and 85, than untreated NLS leaves. At these growth stages, in 2019, no difference was noticed.

No significant differences in Na levels were observed between treated and untreated leaves of the other categories, except for significantly higher contents in treated compared to untreated TIGR leaves, at growth stage 89, in 2019 (Table 7).

Fe contents were similar in the different categories of untreated leaves at the first two growth stages, in both years of study. Later in the season, significantly higher contents in AS, ASTIGR, and TIGR leaves compared to NLS ones, at growth stage 85, in 2020, were noticed (Table S2). Furthermore, in both years, Fe contents of untreated TIGR leaves were significantly higher than that recorded in NLS and AS leaves, at growth stages 89.

Treated vines did not show differences among shoot categories, with the exception of TIGR leaves in 2019 at growth stage 85, with Fe content significantly higher than NLS, AS, and PRE-S leaves (Table S2).

No difference in Fe levels was generally recorded between treated and untreated leaves (Table S2).

In untreated vines, no significant differences in Cu content were noticed among the leaves of the various shoot categories, apart from very few exceptions only related to higher NLS leaf content at growth stage 75 in 2019, in ASTIGR at growth stage 75 in 2020 and in AS at growth stage 85 in 2020 (Table S2).

In treated vines, leaves from the different categories of shoots never differed from each other, except for significantly higher Cu contents in TIGR compared to NLS leaves, at growth stage 85, in 2019 (Table S2).

In the comparison between treated and untreated vines, no differences were generally noticed in Cu content among the different investigated categories (Table S2).

In untreated vines, in 2020, Mn contents were significantly higher: in ASTIGR than PRE-S leaves, at growth stage 75; in AS than NLS, PRE-S and TIGR leaves, at growth stage 85. These results were not observed at the corresponding growth stages in 2019 (Table S3).

No differences in Mn content were found among leaves of treated shoots, except for significantly higher contents in PRE-S than NLS and ASTIGR leaves, at growth stage 85 of 2019 (Table S3).

Mn contents did not show any difference between the leaves of the same shoot categories of treated and untreated vines.

No differences were generally recorded in Zn leaf content (Table S3).

## 3. Discussion

The grapevine trunk diseases (GTDs) are increasing in almost all grape growing areas [43]. Although Esca complex is the most widespread and studied GTDs in European vineyards, some etiological and epidemiological aspects still remained unexplained as well as for the development of an effective control strategy [3]. Losses in quantity and quality of yield in diseased vineyards appeared to be correlated with incidence and severity of plant symptom expression [14,15]. However, mechanisms and factors associated with the occurrence of symptoms are yet to be fully clarified. Therefore, the present study aimed to evaluate some aspects of the physiology of diseased vines involved with the appearance and development of the disease symptom expression.

### 3.1. Macro and Microelements in the Leaf

The analysis of macro and microelements carried out in the present study on samples of leaves collected at different stages of growing season highlighted the dynamics of each element both in never leaf-symptomatic and in the different categories of diseased vine shoots. Therefore, indications have been obtained on a possible role played by these elements in the complex mechanisms of symptom expression.

Levels of calcium and magnesium differed between leaves of never leaf-symptomatic and diseased vines, but also between leaves of different diseased vine shoots. In particular, for most of the growing season, in the parcel not treated with the fertilizer mixture, the leaves of asymptomatic vines, AS, and, at berries pea-sized, the leaves of asymptomatic shoots of symptomatic diseased vines, ASTIGR, had higher levels of calcium and magnesium compared to untreated leaves of never leaf-symptomatic vines, NLS. On the contrary, at pea-sized berries, still green leaves at the base of shoots that began to show symptoms in the leaves of distal part, PRE-S, had lower contents of calcium and magnesium, maybe also due to a shoot malfunction, compared to the leaves of the other shoots, and in particular compared to AS leaves. As observed in a previous study [32], accumulations of calcium, magnesium, sodium, and microelements were recorded in symptomatic leaves, TIGR, probably because of their impaired functionality. Other remarkable differences were in low sodium contents in PRE-S shoot leaves compared to AS leaves, at softening of berries. These results provided a first evidence on the role of calcium and magnesium in the expression of leaf symptoms, notable in both AS and ASTIGR leaves, which remained asymptomatic throughout the season [34,44]. The involvement of calcium and magnesium in the symptom occurrence could be confirmed by low levels of both elements in leaves of PRE-S, compared to AS shoots, which then showed a progressive development of symptoms. Furthermore, the lower sodium contents in PRE-S compared to AS leaves, indicated a possible function of this element in the dynamics of foliar symptom expression. The correlation between sodium and the expression of leaf symptoms is in agreement with what was found in the decay of kiwifruit, a disease similar for many aspects to Esca of grapevine [45].

Fertilizer mixture applications confirmed to be effective in reducing foliar symptom expression [34,44], but also contributed to obtain further information on leaf symptom expression dynamics. Leaves of treated AS vines had higher contents of calcium and magnesium, compared to NLS treated leaves, at berries pea-sized, while at softening of berries, higher calcium and magnesium contents in NLS compared to AS leaves were recorded. AS vines probably responded better to the treatment than NLS vines, because most likely NLS vines did not require calcium and magnesium. Therefore, it may not be excluded that AS vines, due to infection, could have a better physiological attitude to calcium and magnesium supply. Unlike what was observed in untreated AS leaves, treated AS leaves showed sodium dynamics similar to that found for calcium and magnesium, providing further indications of a possible involvement of sodium in foliar symptom expression. Also treated TIGR leaves, as for the untreated, accumulated calcium, magnesium, sodium, and microelements.

The comparison of elements content between treated and untreated leaves gave further information on their dynamics in leaf symptom development. The same dynamics observed for calcium, magnesium, and sodium in treated AS and NLS leaves appeared clearer in the comparison between treated and untreated AS leaves. In fact, contents of the three elements were higher in treated compared to untreated AS leaves, at fruit set and berries pea-sized stages, while at softening of berries contents reversed and were higher in untreated compared to treated AS leaves.

Calcium and magnesium contents were not different between treated and untreated NLS leaves, indicating how the applications of calcium and magnesium, at fruit set and berries pea-sized stages, before the onset of symptoms in the vineyard, were useful in AS vines particularly, by reducing the symptom expression.

Comparisons between leaves of treated and untreated shoots of the same category did generally not show any difference for the other investigated elements.

### 3.2. Role of Calcium in Leaf Symptom Expression

The overall analysis of results on macro and microelements content in leaves of the different categories confirmed the involvement of calcium in the expression of foliar symptoms, hypothesizing a possible role in reducing the symptom expression, as shown in a previous study [34].

The role of calcium was evident both in untreated AS vines, which showed leaf calcium increases for most of the season, and in treated AS vines, whose leaves accumulated calcium up to pea-sized berries, at the onset of symptoms in the vineyard. In treated vines, including the ones who most likely would show symptoms if not treated, calcium accumulations assessed in the first part of the season and due to fertilizer applications, indicated a possible role of the element in the modulation of the plant response, which was hypothesized for leaf symptom expression [46]. This plant response can be associated with phytotoxic substances produced by fungi in infected woody tissues and translocated to the leaf via transpiration current [20,21,47]. Therefore, the development of necrosis in symptomatic leaves was associated with a strong response of the plant to such phytotoxic substances, comparable to a hypersensitive response [48].

To support the hypothesis mentioned, previous studies showed that at pre-bunch closure, tiger-stripe leaves, compared to never leaf-symptomatic and asymptomatic vine leaves, had higher contents of *trans*-resveratrol and other phytoalexins, which are synthesized after the hypersensitive response of the plant [29,31]. 

Furthermore, previous studies demonstrated that vines without foliar symptoms as a consequence of fertilizer mixture applications had higher contents of *trans*-resveratrol, *trans*-ε-viniferin, *trans*-δ-viniferin, and flavonoids, as well as calcium oxalate druse, at pre-bunch closure, with respect to both untreated never leaf-symptomatic and asymptomatic diseased vines [34,35].

In the subsequent growth stages phytoalexins contents progressively decreased in treated vines and increased in untreated vines which showed higher contents than treated vines [35], reproducing the same dynamics of calcium, magnesium, and sodium observed in the present study.

These results strengthened the hypothesis of a defense plant response involved in the foliar symptom expression, indicating as well the role of the fertilizer mixture in this response, by reducing symptoms.

Moreover, what assessed on both untreated and treated asymptomatic plants suggested that calcium activity could be linked to the reduction of plant oxidative response [48]. The penetration of calcium into leaves, facilitated by the seaweed, can increase the synthesis of calmodulin. Calmodulin regulates salicylic acid and, consequently, plant response to fungal toxic metabolites, reducing effects of the hypersensitive response, associated with leaf symptom expression [49].

Moreover, the reduction of symptom expression could also be due to the ability of calcium to strengthen cell walls, as calcium oxalate crystal accumulation, assessed in leaves of treated asymptomatic vines [34,50,51]. Furthermore, the increasing of calcium content in extracellular spaces can increase phytoalexin synthesis [52,53].

### 3.3. Role of Magnesium and Sodium in Leaf Symptom Expression

Applications of the fertilizer mixture were particularly effective in diseased plants, likely for a plant health improvement stimulated by magnesium [54]. It therefore might not be excluded an involvement of magnesium as fundamental constituent of chlorophyll, in reducing or delaying its degradation, limiting as well leaf symptom expression in synergy with calcium [34,44].

The role of magnesium associated with disease control proved to be much more controversial, as it can reduce, but also favor, disease severity [55]. Magnesium can have indirect beneficial effects on diseases with the possibility to withstand disease outbreak, because of the involvement of this element in several physiological processes associated with plant health [54].

In GTDs, studies demonstrated that Mg^++^ and Mn^++^ detoxified eutypin, the toxin produced by *Eutypa lata*, causal agent of Eutypa dieback [56]. It might not be excluded similar effects on toxic metabolites produced by the pathogens involved in Esca of grapevine. Although the reduction of symptom expression was demonstrated in the present study mainly as effect of calcium and magnesium, further studies are needed to give a specific role to magnesium.

The significant sodium increasing at berries pea-sized in treated compared to untreated AS vine leaves could be related to phytoalexin increasing observed in a previous study at the same growth stage. Regulatory mechanisms associated with second messenger metabolites and sodium uptake have been studied; in particular, fast and temporary increases of reactive oxygen species (ROS) involved in hypersensitive response and secondary metabolites synthesis, soon after increases of Na^+^, were recorded [57].

Given the structural similarity of sodium and potassium ions [58], sodium can replace potassium but only in conditions of its deficiency [59,60]. Therefore, a peculiar function of sodium in the leaf symptom expression might even be possible, since the uptake of this element did not seem linked to a leaf potassium deficiency never found in the present study in AS leaves.

### 3.4. Leaf Reflectance Measurements

At berries pea-sized, leaves of never leaf-symptomatic and asymptomatic diseased vines had WI values higher than leaves of the different categories of symptomatic shoots, indicating a higher plant water concentration (PWC). In the first three surveys carried out at full flowering, end of flowering, and fruit set stages, never leaf-symptomatic and asymptomatic diseased vines, treated with the fertilizer mixture, showed WI leaves values higher than those of leaves of corresponding untreated vines. The higher WI values in untreated AS vine leaves, recorded at berries pea-sized, highlighted better physiological efficiency of AS vines, further improved by fertilizer applications.

On the contrary, at full flowering, NDVI and GNDVI values were lower in treated NLS and AS leaves, compared to the untreated ones, while at fruit set, both indices were higher in AS treated compared to untreated, but similar between treated and untreated NLS leaves. These results demonstrated the activity of fertilizer mixture applications carried out until the fruit set. Higher WI and lower NDVI and GNDVI values, observed at full flowering in treated compared to the untreated AS leaves, might be related to the ‘energy’ required for plant defense response modulated by the components of fertilizer mixture.

However, decreasing NDVI and GNDVI values in treated leaves was transitory; in fact, at fruit set, near the beginning of first symptom appearance, these values increased in treated compared to untreated AS leaves, because of an increased photosynthetic activity probably due to fertilizer mixture applications.

At fruit set, higher contents of calcium and magnesium were also recorded on treated compared to untreated AS leaves. In a previous study, at the first days of symptom appearance, *trans*-resveratrol, was higher in treated compared to untreated AS leaves [26]. Thus, the mixture applications seemed to act with a further mechanism of action, by increasing photosynthetic activity, probably favoring increases of the two elements and *trans*-resveratrol.

On the other hand, at berries pea-sized low contents of calcium and magnesium, as well lower values of WI, were detected in untreated PRE-S leaves, confirming the role played by the two elements in limiting foliar symptoms.

## 4. Materials and Methods

### 4.1. Vineyard

Field trials were carried out in a 42-year-old vineyard, cv. Trebbiano d’Abruzzo on 420A rootstock, trained as Geneva Double Courtain (GDC). The vineyard, located in Controguerra (TE), Abruzzo, a central Italy Region, consisted of 700 vines, planted on an area of 5984 m^2^ on a clayey-limestone soil, with a 2 × 4 m planting. The average yield per vine ranged from 13 to 16.5 kg. This vineyard is being surveyed since 1994 for the incidence and severity of tiger-stripe foliar symptoms.

### 4.2. Leaf Applications

Two parcels were identified in the vineyard; one of them was treated with the fertilizer mixture (Algescar ^®^, Natural Development Group, Castelmaggiore, Bologna, Italy) based on CaCl_2_ (33.7%), Mg(NO_3_)_2_ (29.1%), and *Fucales* seaweed extract (6.1%), the second parcel represented the untreated control. Each parcel included three plots of 90 vines, each consisting of a replicate. In 2019 and 2020, vines of a parcel were subjected to six foliar applications with the fertilizer mixture. The mixture was applied with an air blast sprayer at a dose of 5 L, in a water volume of 800 L ha^−1^, at 10–15 days interval, from 53 “inflorescences clearly visible” to 77 “berries beginning to touch” BBCH growth stages [61]. In 2019, applications were performed on 14 May; 3, 12, 21 and 26 June; and 15 July; in 2020 on 13 May; 3, 13, 22 and 26 June; and 15 July.

### 4.3. Leaf Symptom Surveys

The foliar symptom incidence and severity were recorded every year for 25 consecutive seasons. Thus, during the survey of the present study carried out in 2019 and 2020, it was possible to identify the asymptomatic infected vines, which did not show symptoms in the years of survey but have shown symptoms in at least one of the previous years of survey. These asymptomatic vines were distinguished from the never leaf-symptomatic vines, which did not show symptoms in any of the 25 years of survey.

In 2019, four surveys on foliar symptoms were carried out on 8 and 31 July, 27 August and 13 September; in 2020, six surveys were carried out on 7, 20, and 31 July, 10 and 27 August; and 12 September, both in treated and untreated parcels. Surveys in July were performed from 75 “berries pea sized” to 79 “majority of berries touching” BBCH growth stages; in August at 83 “berries developing color” and 85 “softening of berries” stages; in September at 89 “berries ripe for harvest” stage [61].

In each survey, both the disease incidence and severity were recorded on each single plant. The incidence was calculated by dividing the number of plants with symptoms on the total number of diseased vines (that are plants that showed symptoms in at least one of the 25 years of survey) and multiplying by 100. The severity was calculated using the formula SN × 100/(Y × Z), where SN = sum of symptom severity values; Y = number of the monitored plants; Z = maximum value of the symptom scale [62]. The symptom severity was calculated using an arbitrary 0–5 scale, where 0 = absence of symptom; 1 = 1–10%; 2 = 11–30%; 3 = 31–50%; 4 = 51–70%; 5 = 71–100% of foliar symptoms on the canopy.

### 4.4. Leaf Reflectance Measurements

For each plant category (never leaf-symptomatic, diseased but asymptomatic for the entire season and symptomatic diseased) leaf reflectance measurements were carried out in both years of the study on leaves of shoots classified as follows: never leaf-symptomatic (NLS) from never leaf-symptomatic vines and asymptomatic (AS) from diseased but asymptomatic for the entire season vines. In the symptomatic diseased vine group measurements were carried out on: i) pre-symptomatic shoots, namely shoots that showed leaves with early symptoms at the bottom of the shoot, and leaves still without symptoms in the remaining part (PRE-S); ii) shoots with only asymptomatic leaves for the entire season (ASTIGR); and iii) shoots with only symptomatic leaves (TIGR). For each category of shoot, measurements, performed with a portable spectroradiometer mod. Fieldspec Pro^®^, Malvern Panalytical Ltd. (Malvern, UK), were carried out on leaves located in the median part of the primary shoots, opposite to a cluster, in order to avoid variability along the shoot.

The spectroradiometer sensor is equipped with high sensitivity detector array, low straylight, built-in shutter, background current compensation system, and second-order filter that allows detections with a high signal-to-noise ratio in less than a second. Spectrum detection consists in the acquisition of discrete measurements, recorded by the instrument through the internal software and shown on the integrated display. It is then possible to connect the instrument to a computer for processing and exporting the detected spectra, using a special program supplied with the instrument.

For each of categories never leaf-symptomatic vines (NLS) and diseased but asymptomatic for the entire season vines (AS), six plants were identified. In each plant, two shoots were considered, one facing east (side A) and one facing west (side B). In each of the two shoots, five measurements were made on three leaves, finally obtaining an average. Also in the symptomatic diseased vines, six vines were chosen, but for each vine the measurements were performed on three shoots, one for each PRE-S, ASTIGR, and TIGR treatments; in this case, measurements were carried out only on the shoots of side A, given the lack of symptomatic shoots in the part of the canopy facing west (side B).

In NLS and AS vines data of both treated and control parcels were recorded. On the contrary, measurements on the five types of shoot (NLS, AS, PRE-S, ASTIGR, and TIGR) were performed only in the untreated parcel, due to the low expression of foliar symptoms, probably because of the combination of the fertilizer mixture activity and seasonal meteorological conditions.

Reflectance measurements of NLS and AS shoots were carried out in each of the 9 surveys, from 66 “full flowering” to 89 “berries ripe for harvest” BBCH growth stages, whereas measurements of the categories of shoot in the untreated parcel were carried out in six surveys, from 75 “berries pea-sized”, at the onset of symptoms, to 89 “berries ripe for harvest” BBCH growth stages.

NDVI, GNDVI, and WI values were recorded at each reflectance measurement.

### 4.5. Analysis of Macro and Microelements in the Leaf

#### 4.5.1. Leaf Sampling

In order to assess the content of macro and microelements in leaves of the different categories of plants, in 2019 leaf samples were taken on 11 July, 27 August, and 12 September, corresponding respectively to the following BBCH growth stages: 77 “berries beginning to touch”; 85 “softening of berries”; and 89 “berries ripe for harvest”. In 2020, samples were taken on July 9, August 27, and September 12, with an extra sampling on June 27, at 71 “fruit set” [61].

For each plant category (never leaf-symptomatic, diseased but asymptomatic for the entire season and symptomatic diseased) samples were taken on six vines. Eight leaves were taken from each never leaf-symptomatic vine (NLS) and all season asymptomatic diseased vine (AS). From each symptomatic diseased vine, eight leaves were taken for each of the three types of shoot—PRE-S, ASTIGR, and TIGR. For each type of shoot, six samples were collected, each consisting of eight leaves. Leaves were always collected from the median portion of primary shoots and in the opposite position to a cluster.

#### 4.5.2. Macro and Microelements Analysis

##### Reagents and Standards

Mix elements stock standard solution of calcium (Ca), magnesium (Mg), potassium (K), sodium (Na) at 2000, 400, 200 e 1000 mg L^−1^ respectively and single element stock solution of yttrium (Y) at 1000 mg L^−1^ were purchased from Sigma-Aldrich (St. Louis, MO, USA). Mix elements stock standard solution of manganese (Mn), copper (Cu), iron (Fe), zinc (Zn), and single element stock solution of phosphorus (P) at 100 mg L^−1^ were provided by Panreac Química SLU (Castellar del Vallès, Barcelona, Spain) and CPAchem (Stara Zagora, Bulgaria) respectively.

Solutions were prepared with high-purity water of 18.2 MΩ⋅cm resistivity obtained from a PURELAB^®^ (ELGA LabWater, High Wycombe, United Kingdom). Reagents used for the sample digestion were nitric acid at concentration ≥67%, (CHEM-LAB NV, Zedelgem, Belgium), and hydrogen peroxide at 30% (Merck, Darmstadt, Germany). Argon gas of 99.9995% purity was supplied by Sapio (Monza, Italy).

The reference material BCR 1573a, tomato leaves (National Institute of Standard & Technology, Gaithersburg, Maryland, USA) has certified values of concentration of all investigated elements.

##### Analytical Method—Sample Preparation

Each sample was analyzed to determine levels of calcium, magnesium, potassium, sodium, manganese, copper, iron, zinc, and phosphorus.

The sample preparative step was made applying two official methods UNI EN 13804:2013 (sample homogenization) and UNI EN 13805:2014 (sample microwave digestion). 

The instrumental analysis of all investigated elements was conducted by inductively coupled plasma–atomic emission spectrometry (ICP-AES) applying official method UNI EN 15621:2017.

The method provides for a complete destruction of organic matter with nitric acid and hydrogen peroxide at high temperatures and pressure, in a closed vessel, applying microwave assisted heating, prior to ICP-AES analysis.

At each session, a certified reference material (BCR 1573a) as quality control for all investigated elements was analyzed.

Prior to analysis, all the apparatus intended to come into direct contact with the sample and glassware were treated with nitric acid solution (1 ÷ 2%) and then rinsed with high-purity water.

Leaf samples, with own foil and petiole, after sampling in the field, were stored at the temperature of −20 °C.

At the time of analysis, samples were homogenized with cutting mill (Grindomix GM-200, Retsch, Germany) at 9000 rpm for (30 ÷ 60) seconds.

Homogenized sample (500 ± 50) mg L^−1^ was weighed into PTFE vessels and dissolved in 5 mL of concentrated nitric acid and 1 mL of hydrogen peroxide at 30%. Mineralization was performed in a Multiwave 3000 microwave digestion system (Anton Paar, Graz, Austria) according to the program shown in Table 8.

After cooling, the resulting clear solutions (samples, blank and reference material) were quantitatively transferred and diluted exactly to a volume of 15 mL with high-purity water.

Analytical Method—Instrumental Analysis

Analysis of investigated elements was carried out with an inductively coupled plasma atomic emission spectrometer Optima 7000 (PerkinElmer, Waltham, MA, USA). Measurements were performed applying the instrumental conditions mentioned below (Table 9 and Table 10).

Quantitative determination was performed by an external calibration with Y as internal standard. The linearity range of all calibration curves are reported in Table 11.

At each analytical session an aliquot of certified reference material BCR 1573a was analyzed. In Table 12 certified values and achieved recovery average were reported.

### 4.6. Statistical Analysis

In both years of the study, the incidence and severity of foliar symptoms recorded in the parcel treated with the fertilizer mixture, were compared with values of the untreated parcel. The comparison was carried out at harvest (13 September 2019 and 12 September 2020) using Chi-square tests at *p* = 0.05, following what reported in our previous study [25]. A one-way analysis of variance (ANOVA) was applied for each survey or sampling values, respectively of NDVI, GNDVI, WI or macro and microelements, recorded in leaves of NLS and AS vines and in leaves of shoots of symptomatic diseased vines ASTIGR, PRE-S, and TIGR. When significant differences emerged, means separation was performed by Tukey’s honest significant difference (HSD) test at *p* = 0.05. Statistical analysis was performed using XLSTAT 2016 (Addinsoft, Paris, France).

## 5. Conclusions

Dynamics of macro and microelements and vegetation indices highlighted in this study, as for the dynamics of phytoalexins discussed in our recent studies, suggested the role of calcium and magnesium in tiger-stripe foliar symptoms expression. These results also reinforced the hypothesis on the triggering of a complex response of the plant to the occurrence and development of leaf symptoms induced by toxic substances promoted by pathogens.

Calcium could play a role in modulating the plant’s response to toxic fungal metabolites, reducing the effects of an uncontrolled reaction associated with the expression of foliar symptoms in diseased vines.

The activity of calcium was indicated both in untreated vines and in the vines treated with the fertilizer mixture, which significantly reduced the symptom expression, although with different dynamics.

Calcium, magnesium, and sodium contents were particularly high in leaves of treated asymptomatic diseased vines at berries pea-sized, when foliar symptoms began to appear in the vineyard. In these leaves, the high content of the three elements was accompanied by high values of WI, preceded by high NDVI and GNDVI values recorded at fruit set, and followed by an early synthesis of phytoalexins, demonstrated in previous studies. 

Therefore, increased availability of calcium and magnesium up to pea-sized berries reduced foliar symptom expression just when they begin to appear.

Further evidence carried out in different grape growing areas and infection conditions are needed to strengthen the hypothesis discussed in the present study.

## Figures and Tables

**Table 1:** NDVI and GNDVI in leaves of treated and untreated never leaf-symptomatic vines and diseased asymptomatic vines in the side A rows of Controguerra vineyard.

	NDVI								
	66 BBCH	69 BBCH	71 BBCH	75 BBCH	77 BBCH	79 BBCH	83 BBCH	85 BBCH	89 BBCH
Treatment	14/06/2019	21/06/2019	27/06/2019	08/07/2019	16/07/2019	31/07/2019	12/08/2019	27/08/2019	13/09/2019
NLSnt	0.914 a	0.866 a	0.820 ab	0.806 a	0.838 a	0.783 a	0.810 a	0.823 a	0.819 a
ASnt	0.897 a	0.857 a	0.808 b	0.834 a	0.823 a	0.799 a	0.808 a	0.836 a	0.856 a
NLSt	0.804 b	0.867 a	0.798 b	0.788 a	0.842 a	0.826 a	0.814 a	0.844 a	0.844 a
ASt	0.777 b	0.868 a	0.886 a	0.791 a	0.834 a	0.813 a	0.824 a	0.827 a	0.844 a
**Treatment**	**15/06/2020**	**22/06/2020**	**27/06/2020**	**09/07/2020**	**20/07/2020**	**31/07/2020**	**10/08/2020**	**27/08/2020**	**12/09/2020**
NLSnt	0.902 a	0.853 a	0.848 b	0.873 a	0.832 a	0.833 a	0.853 a	0.854 a	0.834 a
ASnt	0.885 a	0.861 a	0.812 b	0.858 a	0.812 a	0.831 a	0.856 a	0.861 a	0.822 a
NLSt	0.798 b	0.859 a	0.833 b	0.830 a	0.856 a	0.848 a	0.855 a	0.831 a	0.806 a
ASt	0.792 b	0.878 a	0.894 a	0.852 a	0.870 a	0.834 a	0.824 a	0.835 a	0.818 a
	**GNDVI**								
**Treatment**	**14/06/2019**	**21/06/2019**	**27/06/2019**	**08/07/2019**	**16/07/2019**	**31/07/2019**	**12/08/2019**	**27/08/2019**	**13/09/2019**
NLSnt	0.727 a	0.624 a	0.596 ab	0.519 a	0.568 a	0.486 a	0.547 a	0.542 a	0.590 a
ASnt	0.698 a	0.590 a	0.551 b	0.567 a	0.540 a	0.477 a	0.533 a	0.549 a	0.673 a
NLSt	0.479 b	0.646 a	0.599 ab	0.532 a	0.608 a	0.574 a	0.571 a	0.539 a	0.651 a
ASt	0.424 b	0.639 a	0.663 a	0.489 a	0.562 a	0.529 a	0.567 a	0.568 a	0.634 a
**Treatment**	**15/06/2020**	**22/06/2020**	**27/06/2020**	**09/07/2020**	**20/07/2020**	**31/07/2020**	**10/08/2020**	**27/08/2020**	**12/09/2020**
NLSnt	0.712 a	0.625 a	0.558 b	0.612 a	0.522 b	0.553 a	0.615 a	0.600 a	0.540 a
ASnt	0.701 a	0.636 a	0.574 b	0.604 a	0.517 b	0.557 a	0.635 a	0.637 a	0.552 a
NLSt	0.542 b	0.667 a	0.648 a	0.632 a	0.685 a	0.658 a	0.654 a	0.642 a	0.511 a
ASt	0.568 b	0.656 a	0.623 a	0.639 a	0.699 a	0.648 a	0.643 a	0.667 a	0.575 a

NDVI = normalized difference vegetation index. GNDVI = green normalized difference vegetation index. NLS = never leaf-symptomatic vine leaves; AS = diseased but asymptomatic for the entire season vine leaves; t = treated with fertilizer mixture; nt = untreated. For each column of each year, values followed by the same letter do not differ statistically according to Tukey’s honest significant difference (HSD) test at *p* = 0.05.

**Table 2:** NDVI and GNDVI in leaves of treated and untreated never leaf-symptomatic vines and diseased asymptomatic vines in the side B rows of Controguerra vineyard.

	NDVI								
	66 BBCH	69 BBCH	71 BBCH	75 BBCH	77 BBCH	79 BBCH	83 BBCH	85 BBCH	89 BBCH
Treatment	14/06/2019	21/06/2019	27/06/2019	08/07/2019	16/07/2019	31/07/2019	12/08/2019	27/08/2019	13/09/2019
NLSnt	0.928 a	0.904 a	0.938 b	0.950 a	0.951 a	0.934 a	0.950 a	0.936 a	0.935 a
ASnt	0.907 a	0.915 a	0.958 b	0.971 a	0.957 a	0.947 a	0.957 a	0.946 a	0.959 a
NLSt	0.810 c	0.934 a	0.978 a	0.974 a	0.967 a	0.927 a	0.913 a	0.944 a	0.950 a
ASt	0.867 b	0.936 a	0.981 a	0.971 a	0.975 a	0.933 a	0.943 a	0.927 a	0.940 a
**Treatment**	**15/06/2020**	**22/06/2020**	**27/06/2020**	**09/07/2020**	**20/07/2020**	**31/07/2020**	**10/08/2020**	**27/08/2020**	**12/09/2020**
NLSnt	0.981 a	0.913 a	0.946 b	0.979 a	0.979 a	0.971 a	0.964 a	0.971 a	0.938 a
ASnt	0.977 a	0.922 a	0.943 b	0.977 a	0.976 a	0.974 a	0.971 a	0.977 a	0.931 a
NLSt	0.838 b	0.945 a	0.984 a	0.965 a	0.942 b	0.969 a	0.970 a	0.974 a	0.948 a
ASt	0.849 b	0.955 a	0.984 a	0.975 a	0.951 b	0.967 a	0.973 a	0.973 a	0.940 a
	**GNDVI**								
**Treatment**	**14/06/2019**	**21/06/2019**	**27/06/2019**	**08/07/2019**	**16/07/2019**	**31/07/2019**	**12/08/2019**	**27/08/2019**	**13/09/2019**
NLSnt	0.764 a	0.713 a	0.794 b	0.764 a	0.787 a	0.737 a	0.799 a	0.735 a	0.757 a
ASnt	0.730 a	0.745 a	0.831 b	0.850 a	0.814 a	0.766 a	0.829 a	0.768 a	0.847 a
NLSt	0.504 b	0.790 a	0.864 b	0.880 a	0.847 a	0.770 a	0.752 a	0.839 a	0.833 a
ASt	0.546 b	0.781 a	0.877 a	0.864 a	0.859 a	0.789 a	0.809 a	0.768 a	0.808 a
**Treatment**	**15/06/2020**	**22/06/2020**	**27/06/2020**	**09/07/2020**	**20/07/2020**	**31/07/2020**	**10/08/2020**	**27/08/2020**	**12/09/2020**
NLSnt	0.812 a	0.823 a	0.865 ab	0.872 a	0.864 a	0.853 a	0.819 b	0.848 a	0.715 a
ASnt	0.807 a	0.845 a	0.833 b	0.864 a	0.859 a	0.855 a	0.845 ab	0.871 a	0.730 a
NLSt	0.622 b	0.812 a	0.921 a	0.878 a	0.838 a	0.878 a	0.868 ab	0.886 a	0.800 a
ASt	0.676 b	0.833 a	0.915 a	0.895 a	0.842 a	0.875 a	0.883 a	0.892 a	0.765 a

NDVI = normalized difference vegetation index. GNDVI = green normalized difference vegetation index. NLS = never leaf-symptomatic vine leaves; AS = diseased but asymptomatic for the entire season vine leaves; t = treated with fertilizer mixture; nt = untreated. For each column of each year values followed by the same letter do not differ statistically according to Tukey’s honest significant difference (HSD) test at *p* = 0.05.

**Table 3:** Water Index in leaves of treated and untreated never leaf-symptomatic vines and diseased asymptomatic vines in the Controguerra vineyard.

	Side A WI								
	66 BBCH	69 BBCH	71 BBCH	75 BBCH	77 BBCH	79 BBCH	83 BBCH	85 BBCH	89 BBCH
Treatment	14/06/2019	21/06/2019	27/06/2019	08/07/2019	16/07/2019	31/07/2019	12/08/2019	27/08/2019	13/09/2019
NLSnt	1.042 b	1.056 b	1.039 c	1.069 ab	1.046 a	1.020 a	1.041 a	1.013 a	1.018 ab
ASnt	1.086 b	1.072 b	1.064 bc	1.091 a	1.049 a	1.036 a	1.065 a	1.028 a	1.037 a
NLSt	1.114 a	1.100 a	1.078 ab	1.048 b	1.011 a	1.027 a	1.041 a	1.000 a	0.995 ab
ASt	1.110 a	1.103 a	1.107 a	1.068 ab	1.086 a	1.007 a	1.046 a	0.995 a	0.987 b
**Treatment**	**15/06/2020**	**22/06/2020**	**27/06/2020**	**09/07/2020**	**20/07/2020**	**31/07/2020**	**10/08/2020**	**27/08/2020**	**12/09/2020**
NLSnt	1.036 b	1.023 b	1.043 b	1.059 a	1.050 a	1.029 b	1.039 a	1.038 a	1.032 ab
ASnt	1.056 b	1.044 b	1.062 b	1.082 a	1.056 a	1.051 ab	1.047 a	1.063 a	1.051 a
NLSt	1.112 a	1.114 a	1.081 ab	1.060 a	1.058 a	1.081 a	1.048 a	1.029 a	1.017 ab
ASt	1.116 a	1.110 a	1.106 a	1.058 a	1.058 a	1.047 b	1.056 a	1.044 a	1.013 b
	**Side B WI**								
**Treatment**	**14/06/2019**	**21/06/2019**	**27/06/2019**	**08/07/2019**	**16/07/2019**	**31/07/2019**	**12/08/2019**	**27/08/2019**	**13/09/2019**
NLSnt	1.054 c	1.084 b	1.102 c	1.153 a	1.116 a	1.053 a	1.112 a	1.043 ab	1.070 ab
ASnt	1.082 bc	1.116 b	1.130 bc	1.199 a	1.131 a	1.080 a	1.175 a	1.091 a	1.107 a
NLSt	1.123 ab	1.166 a	1.257 ab	1.179 a	1.180 a	1.056 a	1.098 a	0.965 b	1.020 ab
ASt	1.140 a	1.180 a	1.286 a	1.140 a	1.193 a	1.033 a	1.096 a	0.969 b	1.007 b
**Treatment**	**15/06/2020**	**22/06/2020**	**27/06/2020**	**09/07/2020**	**20/07/2020**	**31/07/2020**	**10/08/2020**	**27/08/2020**	**12/09/2020**
NLSnt	1.088 b	1.101 b	1.111 b	1.076 a	1.105 a	1.066 b	1.067 b	1.087 b	1.078 a
ASnt	1.098 b	1.086 b	1.157 b	1.129 a	1.111 a	1.142 a	1.129 a	1.186 a	1.129 a
NLSt	1.122 a	1.143 a	1.203 a	1.106 a	1.094 a	1.119 ab	1.104 ab	1.157 ab	1.040 a
ASt	1.151 a	1.148 a	1.208 a	1.106 a	1.089 a	1.144 a	1.127 a	1.102 ab	1.052 a

WI = water index. NLS = never leaf-symptomatic vine leaves; AS = diseased but asymptomatic for the entire season vine leaves; t = treated with fertilizer mixture; nt = untreated. For each column of each year values followed by the same letter do not differ statistically according to Tukey’s honest significant difference (HSD) test at *p* = 0.05.

**Table 4:** NDVI and GNDVI in never leaf-symptomatic vine leaves and in different categories of leaves of diseased vines in the side A rows of Controguerra vineyard.

	NDVI					
	75 BBCH	77 BBCH	79 BBCH	83 BBCH	85 BBCH	89 BBCH
Treatment	08/07/2019	16/07/2019	31/07/2019	12/08/2019	27/08/2019	13/09/2019
NLS	0.806 a	0.838 a	0.783 a	0.810 a	0.823 a	0.819 a
AS	0.834 a	0.823 a	0.799 a	0.808 a	0.836 a	0.856 a
PRE-S	0.806 a	0.844 a	0.812 a	0.786 a	0.830 a	0.821 a
ASTIGR	0.870 a	0.880 a	0.778 a	0.778 a	0.829 a	0.810 a
TIGR	0.723 b	0.622 b	0.570 b	0.759 a	0.636 b	0.635 b
**Treatment**	**09/07/2020**	**20/07/2020**	**31/07/2020**	**10/08/2020**	**27/08/2020**	**12/09/2020**
NLS	0.873 a	0.832 a	0.833 a	0.853 a	0.854 a	0.834 a
AS	0.858 a	0.812 a	0.831 a	0.856 a	0.861 a	0.822 a
PRE-S	0.852 a	0.798 a	0.820 a	0.859 a	0.875 a	0.784 a
ASTIGR	0.875 a	0.806 a	0.825 a	0.831 a	0.833 a	0.816 a
TIGR	0.743 b	0.658 b	0.678 b	0.634 b	0.600 b	0.542 b
	**GNDVI**					
**Treatment**	**08/07/2019**	**16/07/2019**	**31/07/2019**	**12/08/2019**	**27/08/2019**	**13/09/2019**
NLS	0.519 a	0.568 a	0.486 a	0.547 a	0.542 ab	0.590 a
AS	0.567 a	0.540 a	0.477 a	0.533 a	0.549 ab	0.673 a
PRE-S	0.523 a	0.550 a	0.485 a	0.521 a	0.608 a	0.633 a
ASTIGR	0.569 a	0.648 a	0.462 a	0.496 a	0.563 a	0.601 a
TIGR	0.465 b	0.451 b	0.303 b	0.448 a	0.415 b	0.487 b
**Treatment**	**09/07/2020**	**20/07/2020**	**31/07/2020**	**10/08/2020**	**27/08/2020**	**12/09/2020**
NLS	0.612 a	0.522 a	0.553 a	0.615 a	0.600 a	0.540 a
AS	0.604 a	0.517 a	0.557 a	0.635 a	0.637 a	0.552 a
PRE-S	0.522 ab	0.502 a	0.466 ab	0.573 a	0.589 a	0.425 ab
ASTIGR	0.625 a	0.559 a	0.553 a	0.564 a	0.635 a	0.552 a
TIGR	0.448 b	0.505 a	0.392 b	0.402 b	0.424 b	0.365 b

NDVI = normalized difference vegetation index. GNDVI = green normalized difference vegetation index. NLS = never leaf-symptomatic vine leaves; AS = diseased but asymptomatic for the entire season vine leaves; PRE-S = pre-symptomatic shoot leaves of symptomatic vines; ASTIGR = asymptomatic shoot leaves of symptomatic vines; TIGR = tiger-striped shoot leaves of symptomatic vines. For each column of each year values followed by the same letter do not differ statistically according to Tukey’s honest significant difference (HSD) test at *p* = 0.05.

**Table 5:** Water Index in never leaf-symptomatic vine leaves and in different categories of leaves of diseased vines in the side A rows of Controguerra vineyard.

	WI					
	75 BBCH	77 BBCH	79 BBCH	83 BBCH	85 BBCH	89 BBCH
Treatment	08/07/2019	16/07/2019	31/07/2019	12/08/2019	27/08/2019	13/09/2019
NLS	1.069 ab	1.046 a	1.020 a	1.041 ab	1.013 a	1.018 a
AS	1.091 a	1.049 a	1.036 a	1.065 a	1.028 a	1.037 a
PRE-S	1.059 b	1.057 a	1.027 a	1.020 bc	1.023 a	1.017 a
ASTIGR	1.008 c	1.108 a	1.013 a	0.987 c	1.020 a	1.004 a
TIGR	1.001 c	1.033 a	1.013 a	0.983 c	1.013 a	1.003 a
**Treatment**	**09/07/2020**	**20/07/2020**	**31/07/2020**	**10/08/2020**	**27/08/2020**	**12/09/2020**
NLS	1.059 a	1.050 a	1.029 ab	1.039 a	1.038 ab	1.032 ab
AS	1.082 a	1.056 a	1.051 a	1.047 a	1.063 a	1.051 a
PRE-S	1.010 b	1.033 a	0.995 b	1.020 a	1.028 abc	1.011 b
ASTIGR	1.021 b	1.048 a	0.995 b	1.011 a	1.007 bc	1.012 b
TIGR	1.009 b	1.042 a	1.001 b	1.002 a	0.993 c	1.002 b

WI = water index. NLS = never leaf-symptomatic vine leaves; AS = diseased but asymptomatic for the entire season vine leaves; PRE-S = pre-symptomatic shoot leaves of symptomatic vines; ASTIGR = asymptomatic shoot leaves of symptomatic vines; TIGR = tiger-striped shoot leaves of symptomatic vines. For each column of each year values followed by the same letter do not differ statistically according to Tukey’s honest significant difference (HSD) test at *p* = 0.05.

**Table 6:** Calcium and magnesium content in never leaf-symptomatic vine leaves and in different categories of leaves of diseased vines.

	BBCH 71	BBCH 75		BBCH 85		BBCH 89	
	27/06/2020	11/07/2019	09/07/2020	27/08/2019	27/08/2020	12/09/2019	12/09/2020
Treatment	Calcium (mg g^−1^)						
NLSnt	5.98 bc	4.62 cd	4.80 de	7.67 c	7.23 c	12.21 ab	7.93 b
NLSt	6.37 ab	5.38 bc	5.27 cde	10.73 abc	7.80 bc	6.92 bc	9.58 ab
ASnt	5.15 c	7.06 b	6.23 bc	12.58 ab	10.53 a	12.51 ab	7.73 b
ASt	7.02 a	9.49 a	7.67 d	8.74 c	7.55 c	8.03 bc	9.87 ab
PRE-Snt	n.p.	3.22 d	3.98 e	10.28 abc	10.57 a	11.94 ab	9.75 ab
PRE-St	n.p.	n.p.	n.p.	9.18 bc	8.28 abc	12.00 ab	9.53 ab
ASTIGRnt	n.p.	6.59 b	6.7 bc	11.13 abc	9.63 abc	12.82 a	7.07 b
ASTIGRt	n.p.	n.p.	n.p.	9.38 bc	8.77 abc	13.71 a	8.42 ab
TIGRnt	n.p.	8.91 a	10.03 a	13.17 a	10.07 ab	14.03 a	10.10 ab
TIGRt	n.p.	n.p.	n.p.	13.17 a	10.42 a	13.73 a	11.48 a
**Treatment**	**Magnesium (mg g^−1^)**						
NLSnt	0.76 ab	0.73 cd	0.66 cd	1.21 bc	0.90 b	1.65 a	1.01 abc
NLSt	0.85 a	1.01 bc	1.13 a	1.52 bc	1.56 a	1.57 a	1.35 a
ASnt	0.63 b	1.27 b	0.76 c	1.73 b	1.03 b	1.62 a	0.86 bc
ASt	0.86 a	1.66 a	1.01 ab	1.35 bc	0.87 b	1.14 a	1.22 ab
PRE-Snt	n.p.	0.55 d	0.57 d	1.10 c	1.07 b	1.39 a	1.23 ab
PRE-St	n.p.	n.p.	n.p.	1.41 bc	0.98 b	1.92 a	0.99 abc
ASTIGRnt	n.p.	0.77 cd	0.68 cd	1.41 bc	0.96 b	1.63 a	0.71 c
ASTIGRt	n.p.	n.p.	n.p.	1.48 bc	1.16 ab	2.05 a	1.09 ab
TIGRnt	n.p.	1.00 bc	0.93 b	1.57 bc	1.00 b	1.55 a	1.01 abc
TIGRt	n.p.	n.p.	n.p.	2.35 a	1.23 ab	1.45 a	1.18 ab

NLS = never leaf-symptomatic vine leaves; AS = asymptomatic diseased vine leaves; PRE-S = pre-symptomatic leaves of symptomatic vines; ASTIGR = asymptomatic leaves of symptomatic vines; TIGR = tiger-striped leaves of symptomatic vines. t = treated with fertilizer mixture; nt = untreated; n.p. = not present category. For each column of each year values followed by the same letter do not differ statistically according to Tukey’s honest significant difference (HSD) test at *p* = 0.05.

**Table 7:** Sodium content in never leaf-symptomatic vine leaves and in different categories of leaves of diseased vines.

	BBCH 71	BBCH 75		BBCH 85		BBCH 89	
	27/06/2020	11/07/2019	09/07/2020	27/08/2019	27/08/2020	12/09/2019	12/09/2020
Treatment	Sodium (mg Kg^−1^)						
NLSnt	32.8 b	112.6 a	57.7 c	106.3 ab	64.2 de	84.3 d	72.5 a
NLSt	52.7 a	83.3 ab	110.0 a	86.5 ab	127.0 a	180.0 ab	113.3 a
ASnt	29.5 b	53.7 bc	61.6 bc	111.7 ab	101.7 abcd	135.5 bcd	109.7 a
ASt	55.5 a	112.4 a	124.0 a	90.5 ab	73.5 cde	112.0 bcd	111.2 a
PRE-Snt	n.p.	34.2 c	72.3 bc	55.8 b	60.8 e	100.2 cd	91.5 a
PRE-St	n.p.	n.p.	n.p.	134.5 ab	85.3 bcde	168.3 bc	91.7 a
ASTIGRnt	n.p.	28.3 c	68.7 bc	108.6 ab	66.2 de	87.5 d	67.3 a
ASTIGRt	n.p.	n.p.	n.p.	120.7 ab	76.0 cde	106.2 bcd	71.7 a
TIGRnt	n.p.	35.6 bc	96.3 ab	147.5 a	108.0 abc	102.3 bcd	138.3 a
TIGRt	n.p.	n.p.	n.p.	99.4 ab	117.3 ab	250.2 a	134.8 a

NLS = never leaf-symptomatic vine leaves; AS = asymptomatic diseased vine leaves; PRE-S = pre-symptomatic leaves of symptomatic vines; ASTIGR = asymptomatic leaves of symptomatic vines; TIGR = tiger-striped leaves of symptomatic vines. t = treated with fertilizer mixture; nt = untreated; n.p. = not present category. For each column of each year values followed by the same letter do not differ statistically according to Tukey’s honest significant difference (HSD) test at *p* = 0.05.

**Table 8:** Instrumental parameters of microwave digestion oven.

Step	Power (watt)	Ramp (°C/min)	Duration (min)
1	450	1	4
2	800	5	8
3	1000	5	15
4	0	-	15

**Table 9:** Instrumental conditions of ICP-AES.

**Parameter**	**Value**
Power radiofrequency (W)	1300 ÷ 1500
Nebulizer gas flow (L min^−1^)	0.6 ÷ 0.7
Plasma gas flow (L min^−1^)	15
Auxiliary gas flow (L min^−1^)	0.2
Nebulizer	Mira Mist (in peek)
Spray chamber	Cyclonic

**Table 10:** Wavelength and mode view.

Element	Wavelenght (nm)	Plasma Mode View
Fe	238.204	Axial
Cu	327.393	
Zn	213.857	
Mn	257.610	Attenuated Axial
P	213.617	Radial
Na	589.592	
Ca	317.933	
Mg	285.213	
K	766.490	
Y	361.104–371.029	-

**Table 11:** Linearity range of calibration curve of elements.

Element	Linearity Range (mg L^−1^)
Fe	0.0050 ÷ 1.0
Cu	
Zn	
Mn	
P	0.25 ÷ 10
Na	0.13 ÷ 2.0
Ca	0.25 ÷ 10
Mg	0.050 ÷ 2.0
K	0.025 ÷ 1.0

**Table 12:** Certified values of BCR1573a and achieved recovery average.

Element	Certified Values (mg Kg^−1^)	Recovery (%) (n = 5)
Fe	368 ± 7	100
Cu	4.70 ± 0.14	109
Zn	30.9 ± 0.7	97
Mn	246 ± 8	98
P *	0.216 ± 0.004	102
Na	136 ± 4	95
Ca *	5.05 ± 0.09	101
Mg *	1.2 **	93
K *	2.70 ± 0.05	91

* measurement unit (%); ** information value; n = number of analytical session.

## Data Availability

Data are reported within the Article and Supplementary materials.

## Supplementary Materials

The following are available online at https://www.mdpi.com/article/10.3390/plants10061041/s1, Table S1: Phosphorus and potassium content in never leaf-symptomatic vine leaves and in different categories of leaves of diseased vines, Table S2: Iron and Copper content in never leaf-symptomatic vine leaves and in different categories of leaves of diseased vines, Table S3: Manganese and Zinc content in never leaf-symptomatic vine leaves and in different categories of leaves of diseased vines.

## References

[1] Mugnai L., Graniti A., Surico G. (1999). Esca (black measles) and brown wood streaking: Two old and elusive diseases of grapevines. Plant Dis..

[2] Bertsch C., Ramírez-Suero M., Magnin-Robert M., Larignon P., Chong J., Abou-Mansour E., Spagnolo A., Clèment C., Fontaine F. (2013). Grapevine trunk diseases: Complex and still poorly understood. Plant Pathol..

[3] Gramaje D., Urbez-Torres J.R., Sosnowski M.R. (2018). Managing grapevine trunk diseases with respect to etiology and epidemiology: Current strategies and future prospects. Plant Dis..

[4] Brown A.A., Lawrence D.P., Baumgartner K. (2020). Role of basidiomycete fungi in the grapevine trunk disease esca. Plant Pathol..

[5] Baránek M., Armengol J., Holleinová V., Pečenka J., Calzarano F., Peňázová E., Vachůn M., Eichmeier A. (2018). Incidence of symptoms and fungal pathogens associated with grapevine trunk diseases in Czech vineyards: First example from a north-eastern European grape-growing region. Phytopathol. Mediterr..

[6] Surico G. (2009). Towards a redefinition of the diseases within the esca complex. Phytopathol. Mediterr..

[7] Fischer M. (2006). Biodiversity and geographic distribution of basidiomycetes causing esca-associated white rot in grapevine: A worldwide perspective. Phytopathol. Mediterr..

[8] Edwards J., Marchi G., Pascoe I. (2001). Young esca in Australia. Phytopathol. Mediterr..

[9] Calzarano F., Di Marco S. (2007). Wood discoloration and decay in grapevines with esca proper and their relationship with foliar symptoms. Phytopathol. Mediterr..

[10] Lecomte P., Darrieutort G., Liminana J.M., Louvet G., Tandonnet J.P. (2008). Eutypiose et esca: I: Eléments de réflexion pour mieux appréhender ces phénomènes de dépérissement. Phytoma.

[11] Ouadi L., Bruez E., Bastien S., Vallance J., Lecomte P., Domec J.C., Rey P. (2019). Ecophysiological impacts of Esca, a devastating grapevine trunk disease, on *Vitis vinifera* L. PloS ONE.

[12] Maher N., Piot J., Bastien S., Vallance J., Rey P., Guérin-Dubrana L. (2012). Wood necrosis in esca-affected vines: Types, relationships and possible links with foliar symptom expression. J. Int. Sci. Vigne Vin..

[13] Bruez E., Vallance J., Gerbore J., Lecomte P., Da Costa J.P., Guerin-Dubrana L., Rey P. (2014). Analyses of the temporal dynamics of fungal communities colonizing the healthy wood tissues of esca leaf-symptomatic and asymptomatic vines. PLoS ONE.

[14] Calzarano F., Seghetti L., Del Carlo M., Cichelli A. (2004). Effect of esca on the quality of berries, musts and wines. Phytopathol. Mediterr..

[15] Lorrain B., Ky I., Pasquier G., Jourdes M., Guerin Dubrana L., Gény L., Rey P., Donèche B., Teissedre P.L. (2012). Effect of Esca disease on the phenolic and sensory attributes of Cabernet Sauvignon grapes, musts and wines. Aust. J. Grape Wine R..

[16] Marchi G., Peduto F., Mugnai L., Di Marco S., Calzarano F., Surico G. (2006). Some observations on the relationship on manifest and hidden esca to rainfall. Phytopathol. Mediterr..

[17] Calzarano F., Osti F., Baránek M., Di Marco S. (2018). Rainfall and temperature influence expression of foliar symptoms of grapevine leaf stripe disease (esca complex) in vineyards. Phytopathol. Mediterr..

[18] Lecomte P., Diarra B., Carbonneau A., Rey P., Chevrier C. (2018). Esca of grapevine and training practices in France: Results of a 10-year survey. Phytopathol. Mediterr..

[19] Sparapano L., Bruno G., Graniti A. (2000). Effects on plants of metabolites produced in culture by *Phaeoacremonium chlamydosporum*, *P. aleophilum* and *Fomitiporia punctata*. Phytopathol. Mediterr..

[20] Evidente A., Sparapano L., Andolfi A., Bruno G. (2000). Two naphthalenone pentaketides from liquid cultures of *Phaeoacremonium aleophilum*, a fungus associated with esca of grapevine. Phytopathol. Mediterr..

[21] Tabacchi R., Fkyerat A., Poliart C., Dubin G. (2000). Phytotoxins from fungi of esca of grapevine. Phytopathol. Mediterr..

[22] Andolfi A., Mugnai L., Luque J., Surico G., Cimmino A., Evidente A. (2011). Phytotoxins produced by fungi associated with grapevine trunk diseases. Toxins.

[23] Magnin-Robert M., Letousey P., Spagnolo A., Rabenoelina F., Jacquens L., Mercier L., Clément C., Fontaine F. (2011). Leaf stripe form of esca induces alteration of photosynthesis and defence reactions in presymptomatic leaves. Funct. Plant Biol..

[24] Claverie M., Notaro M., Fontaine F., Wery J. (2020). Current knowledge on Grapevine Trunk Diseases with complex etiology: A systemic approach. Phytopathol. Mediterr..

[25] Lecomte P., Darrieutort G., Liminana J.M., Comont G., Muruamendiaraz A., Legorburu F.J., Choueiri E., Jreijiri F., El Amil R., Fermaud M. (2012). New insights into esca of grapevine: The development of foliar symptoms and their association with xylem discoloration. Plant Dis..

[26] Bortolami G., Gambetta G.A., Delzon S., Lamarque L.J., Pouzoulet J., Badel E., Burlett R., Charrier G., Cochard H., Dayer S. (2019). Exploring the hydraulic failure hypothesis of esca leaf symptom formation. Plant Physiol..

[27] Pouzoulet J., Scudiero E., Schiavon M., Santiago L.S., Rolshausen P.E. (2019). Modeling of xylem vessel occlusion in grapevine. Tree Physiol..

[28] Larignon P. (2017). Effect of sodium arsenite on the life cycles of the pathogenic agents involved in wood grapevine diseases. Phytopathol. Mediterr..

[29] Calzarano F., D’Agostino V., Pepe A., Osti F., Della Pelle F., de Rosso M., Flamini R., Di Marco S. (2016). Patterns of phytoalexins in the grapevine leaf stripe disease (esca complex)/grapevine pathosystem. Phytopathol. Mediterr..

[30] Calzarano F., Osti F., D’Agostino V., Pepe A., Della Pelle F., de Rosso M., Flamini R., Di Marco S. (2017). Levels of phytoalexins in vine leaves with different degrees of grapevine leaf stripe disease symptoms (Esca complex of diseases). Phytopathol. Mediterr..

[31] Heath M.C. (2000). Hypersensitive response-related death. Plant Mol. Biol..

[32] Calzarano F., Amalfitano C., Seghetti L., Cozzolino V. (2009). Nutritional status of vines affected with esca proper. Phytopathol. Mediterr..

[33] Di Marco S., Osti F. (2009). Effect of biostimulant sprays on *Phaeomoniella chlamydospora* and esca proper infected vines under greenhouse and field conditions. Phytopathol. Mediterr..

[34] Calzarano F., Di Marco S., D’Agostino V., Schiff S., Mugnai L. (2014). Grapevine leaf stripe disease (esca complex) are reduced by a nutrients and seaweed mixture. Phytopathol. Mediterr..

[35] Calzarano F., Osti F., D’Agostino V., Pepe A., Di Marco S. (2017). Mixture of calcium, magnesium and seaweed affects leaf phytoalexin contents and grape ripening on vines with grapevine leaf stripe disease. Phytopathol. Mediterr..

[36] Bhakta I., Phadikar S., Majumder K. (2019). State-of-the-art technologies in precision agriculture: A systematic review. J. Sci. Food Agric..

[37] Vincini M., Calegari F., Casa R. (2015). Sensitivity of leaf chlorophyll empirical estimators obtained at Sentinel-2 spectral resolution for different canopy structures. Precis. Agric..

[38] Wójtowicz M., Wójtowicz A., Piekarczyk J. (2016). Application of remote sensing methods in agriculture. Commun. Biom. Crop. Sci..

[39] Daughtry C. (2000). Estimating corn leaf chlorophyll concentration from leaf and canopy reflectance. Remote Sens. Environ..

[40] Rouse J.W., Haas R.H., Schell J.A., Deering D.W., Freden S.C., Mercanti E.P., Becker M. (1974). Monitoring Vegetation Systems in the Great Plains with ERTS. Third Earth Resources Technology Satellite–1 Symposium.

[41] Gitelson A.A., Kaufman Y., Merzlyak M.N. (1996). Use of a green channel in remote sensing of global vegetation from EOS-MODIS. Remote Sens. Environ..

[42] Penuelas J., Pinol J., Ogaya R., Filella I. (1997). Estimation of plant water concentration by the reflectance Water Index WI (R900/R970). Int. J. Remote Sens..

[43] Mondello V., Songy A., Battiston E., Pinto C., Coppin C., Trotel-Aziz P., Clément C., Mugnai L., Fontaine F. (2018). Grapevine trunk diseases: A review of fifteen years of trials for their control with chemicals and biocontrol agents. Plant Dis..

[44] Calzarano F., Di Marco S. (2018). Further evidence that calcium, magnesium and seaweed mixtures reduce grapevine leaf stripe symptoms and increase grape yield. Phytopathol. Mediterr..

[45] Di Marco S., Osti F. (2008). Foliar symptom expression of wood decay in *Actinidia deliciosa* in relation to environmental factors. Plant Dis..

[46] Lecourieux D., Ranjeva R., Pugin A. (2006). Calcium in plant defence-signalling pathways. New Phytol..

[47] Andolfi A., Cimmino A., Evidente A., Iannaccone M., Capparelli R., Mugnai L., Surico G. (2009). A new flow cytometry technique to identify *Phaeomoniella chlamydospora* exopolysaccharides and study mechanisms of esca grapevine foliar symptoms. Plant Dis..

[48] Lima M.R.M., Ferreres F., Dias A.C.P. (2012). Response of *Vitis vinifera* cell cultures to *Phaeomoniella chlamydospora*: Changes in phenolic production, oxidative state and expression of defence-related genes. Eur. J. Plant Pathol..

[49] Du L., Ali G.S., Simons K.A., Hou J., Yang T., Reddy A.S.N., Poovaiah B.W. (2009). Ca^2+^/ calmodulin regulate salicylic-acid-mediated plant immunity. Nature.

[50] Conway W.S., Sams C.E., Abbott J.A., Bruton B.D. (1991). Postharvest calcium treatment of apple fruit to provide broad-spectrum protection against postharvest pathogens. Plant Dis..

[51] Kratzke M.G. (1988). Study of Mechanism of Calcium Uptake by Potato Tubers and of Cellular Properties Affecting Soft Rot. Ph.D. Thesis.

[52] Stäb M.R., Ebel J. (1987). Effects of Ca^2+^ on phytoalexin induction by fungal elicitor in soybean cells. Arch. Biochem. Biophys..

[53] Tavernier E., Wendehenne D., Blein J.P., Pugin A. (1995). Involvement of free calcium in action of cryptogein, a proteinaceous elicitor of hypersensitive reaction in tobacco cells. Plant Physiol..

[54] Datnoff L.E., Elmer W.H., Huber D.M. (2007). Mineral Nutrition and Plant Disease.

[55] Huber D.M., Jones J.B. (2013). The role of magnesium in plant disease. Plant Soil.

[56] Colrat S., Deswarte C., Latché A., Klaébe K., Bouzayen M., Fallot J., Roustan J.P. (1999). Enzymatic detoxification of eutypine, a toxin from *Eutypa lata*, by *Vitis vinifera* cells: Partial purification of an NADPH-dependent aldehyde reductase. Planta.

[57] Maathuis F.J. (2014). Sodium in plants: Perception, signalling, and regulation of sodium fluxes. J. Exp. Bot..

[58] Amtmann A., Sanders D. (1999). Mechanisms of Na (+) uptake by plant cells. Adv. Bot. Res..

[59] Fageria V.D. (2001). Nutrient interactions in crop plants. J. Plant Nutr..

[60] Garciadeblas B., Senn M.E., Banuelos M.A., Rodriguez-Navarro A. (2003). Sodium transport and HKT transporters: The rice model. Plant J..

[61] Lorenz D.H., Eichhorn K.W., Bleiholder H., Close R., Meier U., Weber E. (1995). Phenological growth stages of the grapevine (*Vitis vinifera* L. ssp. vinifera). Encoding and description of the phenological stages of the grapevine according to the extended BBCH scheme. Aust. J. Grape Wine Res..

[62] McKinney H.H. (1923). Influence of soil temperature and moisture on infection of wheat seedlings by *Helminthosporium sativum*. J. Agr. Res..

