# Supplementary material for: Factors Involved on Tiger-Stripe Foliar Symptom Expression of Esca of Grapevine
Source: Plants (Basel). 2021 May 21;10(6):1041. doi: 10.3390/plants10061041 (PMC8224345; doi:10.3390/plants10061041)
Supplement: Supplementary file 1 [file plants-10-01041-s001.zip › plants-1178059-supplementary.pdf]

Table S1. Phosphorus and potassium content in never leaf-symptomatic vine leaves and in different categories of leaves of diseased vines.

	**BBCH 71**	**BBCH 75**		**BBCH 85**		**BBCH 89**	
	**27/06/2020**	**11/07/2019**	**09/07/2020**	**27/08/2019**	**27/08/2020**	**12/09/2019**	**12/09/2020**
**Treatment**	**Phosphorus (mg g^{-1})**						
NLSnt	0.58 a	0.49 a	0.30 b	0.82 ab	0.45 c	0.59 bc	0.44 c
NLSt	0.51 a	0.40 a	0.33 b	0.53 b	0.60 abc	0.61 bc	0.55 bc
ASnt	0.54 a	0.53 a	0.59 a	1.04 a	0.61 abc	1.16 a	0.73 a
ASt	0.49 a	0.44 a	0.31 b	0.50 b	0.41 c	0.41 c	0.59 abc
PRE-Snt	n.p.	0.47 a	0.59 a	0.54 b	0.68 ab	0.71 bc	0.75 a
PRE-St	n.p.	n.p.	n.p.	0.57 b	0.54 bc	0.60 bc	0.69 ab
ASTIGRnt	n.p.	0.44 a	0.31 b	0.81 ab	0.79 a	0.61 bc	0.54 bc
ASTIGRt	n.p.	n.p.	n.p.	0.51 b	0.54 bc	0.53 c	0.49 c
TIGRnt	n.p.	0.47 a	0.65 a	0.96 a	0.76 ab	0.59 bc	0.72 a
TIGRt	n.p.	n.p.	n.p.	0.49 b	0.72 ab	0.86 ab	0.68 ab
**Treatment**	**Potassium (mg g^{-1})**						
NLSnt	2.45 a	3.17 ab	2.62 a	2.71 a	2.00 cd	4.02 bc	2.17 abcd
NLSt	2.16 a	2.27 b	2.48 a	3.27 a	1.92 d	2.78 de	1.66 cd
ASnt	2.30 a	3.36 ab	2.93 a	3.43 a	2.42 abcd	5.22 a	1.93 abcd
ASt	2.07 a	2.68 ab	2.37 a	3.07 a	2.15 cd	2.75 de	2.38 ab
PRE-Snt	n.p.	3.65 a	3.02 a	3.32 a	2.62 abc	3.38 bcd	2.00 abcd
PRE-St	n.p.	n.p.	n.p.	2.62 a	2.28 bcd	2.95 cde	1.85 bcd
ASTIGRnt	n.p.	3.13 ab	2.62 a	3.03 a	2.90 ab	4.33 ab	1.53 d
ASTIGRt	n.p.	n.p.	n.p.	2.99 a	2.30 abcd	3.48 bcd	1.95 abcd
TIGRnt	n.p.	3.65 a	2.45 a	3.52 a	2.93 a	2.53 de	2.25 abc
TIGRt	n.p.	n.p.	n.p.	2.58 a	2.57 abc	1.78 e	2.53 a

NLS = never leaf-symptomatic vine leaves; AS = asymptomatic diseased vine leaves; PRE-S = pre-symptomatic leaves of symptomatic vines; ASTIGR = asymptomatic leaves of symptomatic vines; TIGR = tiger-striped leaves of symptomatic vines. t = treated with fertilizer mixture; nt = untreated; n.p. = not present category. For each column of each year, values followed by the same letter do not differ statistically according to Tukey's honest significant difference (HSD) test at $p = 0.05$.

Table S2. Iron and Copper content in never leaf-symptomatic vine leaves and in different categories of leaves of diseased vines.

	BBCH 71	BBCH 75		BBCH 85		BBCH 89	
	**27/06/2020**	**11/07/2019**	**09/07/2020**	**27/08/2019**	**27/08/2020**	**12/09/2019**	**12/09/2020**
**Treatment**				**Iron (mg Kg^{-1})**			
NLSnt	16.8 b	43.1 a	22.3 a	65.0 b	23.5 c	57.3 b	28.8 bc
NLSt	21.8 a	35.0 a	29.0 a	62.7 b	29.8 bc	71.8 b	46.3 ab
ASnt	17.7 b	39.3 a	27.2 a	59.0 b	43.2 a	74.5 b	23.7 c
ASt	19.7 ab	43.4 a	31.7 a	67.7 b	33.0 abc	78.8 b	39.7 abc
PRE-Snt	n.p.	44.6 a	26.0 a	64.3 b	35.5 abc	58.2 b	35.3 abc
PRE-St	n.p.	n.p.	n.p.	67.3 b	27.5 bc	65.3 b	34.2 abc
ASTIGRnt	n.p.	34.9 a	27.8 a	67.0 b	39.5 ab	94.7 ab	32.3 abc
ASTIGRt	n.p.	n.p.	n.p.	69.8 ab	36.5 ab	67.0 b	34.5 abc
TIGRnt	n.p.	35.4 a	23.2 a	82.3 ab	37.5 ab	130.6 ab	50.3 a
TIGRt	n.p.	n.p.	n.p.	95.2 a	37.5 ab	94.5 ab	38.7 abc
**Treatment**				**Copper (mg Kg^{-1})**			
NLSnt	154.1 a	178.7 a	215.0 bc	224.5 ab	344.3 b	225.6 b	377.3 a
NLSt	133.8 a	118.00 ab	268.0 abc	162.8 b	446.2 ab	208.5 b	413.2 a
ASnt	178.7 a	83.7 ab	275.5 abc	209.8 ab	556.5 a	225.8 b	370.0 a
ASt	143.5 a	159.8 ab	323.2 ab	217.8 ab	345.3 b	170.2 b	348.2 a
PRE-Snt	n.p.	52.8 b	186.2 c	187.3 b	307.3 b	149.5 b	349.2 a
PRE-St	n.p.	n.p.	n.p.	251.2 ab	388.0 ab	188.3 b	257.0 a
ASTIGRnt	n.p.	52.0 b	341.5 a	262.2 ab	405.5 ab	204.7 b	445.0 a
ASTIGRt	n.p.	n.p.	n.p.	241.6 ab	434.8 ab	138.4 b	342.8 a
TIGRnt	n.p.	54.9 b	206.5 c	232.8 ab	366.0 b	367.5 ab	330.4 a
TIGRt	n.p.	n.p.	n.p.	385.5 a	427.0 ab	274.7 ab	333.8 a

NLS = never leaf-symptomatic vine leaves; AS = asymptomatic diseased vine leaves; PRE-S = pre-symptomatic leaves of symptomatic vines; ASTIGR = asymptomatic leaves of symptomatic vines; TIGR = tiger-striped leaves of symptomatic vines. t = treated with fertilizer mixture; nt = untreated; n.p. = not present category. For each column of each year, values followed by the same letter do not differ statistically according to Tukey's honest significant difference (HSD) test at p = 0.05.

Table S3. Manganese and Zinc content in never leaf-symptomatic vine leaves and in different categories of leaves of diseased vines.

	BBCH 71	BBCH 75		BBCH 85		BBCH 89	
	27/06/2020	11/07/2019	09/07/2020	27/08/2019	27/08/2020	12/09/2019	12/09/2020
**Treatment**	**Manganese (mg Kg^{-1})**						
NLSnt	35.3 a	89.6 a	34.8 ab	53.8 ab	31.5 b	66.2 a	34.0 ab
NLSt	39.0 a	60.5 a	45.5 ab	44.5 b	29.3 b	66.3 a	44.1 ab
ASnt	35.6 a	70.0 a	32.6 ab	56.1 ab	51.7 a	85.0 a	26.3 b
ASt	42.5 a	89.2 a	52.0 a	62.3 ab	40.3 ab	56.5 a	38.4 ab
PRE-Snt	n.p.	50.7 a	21.4 b	49.1 ab	35.5 b	46.2 a	38.6 ab
PRE-St	n.p.	n.p.	n.p.	78.8 a	37.3 ab	59.5 a	32.7 ab
ASTIGRnt	n.p.	60.8 a	57.8 a	59.0 ab	39.7 ab	66.8 a	39.3 ab
ASTIGRt	n.p.	n.p.	n.p.	44.2 b	34.1 b	60.0 a	47.6 a
TIGRnt	n.p.	59.7 a	40.0 ab	71.0 ab	34.5 b	62.2 a	36.3 ab
TIGRt	n.p.	n.p.	n.p.	58.3 ab	42.5 ab	79.3 a	48.7 a
**Treatment**	**Zinc (mg Kg^{-1})**						
NLSnt	9.8 a	21.3 a	9.02 ab	13.8 a	7.8 b	18.0 ab	8.0 abcd
NLSt	9.9 a	14.7 a	10.2 a	16.1 a	11.03 a	12.3 b	8.1 abcd
ASnt	9.6 a	17.1 a	8.1 ab	14.5 a	9.07 ab	15.5 ab	7.7 bcd
ASt	10.7 a	18.9 a	9.2 ab	19.3 a	7.52 b	15.1 ab	9.9 abc
PRE-Snt	n.p.	13.3 a	6.8 b	14.5 a	11.43 a	14.2 ab	10.3 a
PRE-St	n.p.	n.p.	n.p.	18.2 a	9.18 ab	12.7 b	9.0 abcd
ASTIGRnt	n.p.	14.1 a	9.2 ab	15.1 a	8.95 ab	16.0 ab	7.6 cd
ASTIGRt	n.p.	n.p.	n.p.	15.8 a	8.60 ab	12.6 b	6.9 d
TIGRnt	n.p.	13.3 a	9.3 a	17.7 a	9.25 ab	20.3 a	10.1 ab
TIGRt	n.p.	n.p.	n.p.	18.3 a	10.62 ab	13.0 ab	10.1 ab

NLS = never leaf-symptomatic vine leaves; AS = asymptomatic diseased vine leaves; PRE-S = pre-symptomatic leaves of symptomatic vines; ASTIGR = asymptomatic leaves of symptomatic vines; TIGR = tiger-striped leaves of symptomatic vines. t = treated with fertilizer mixture; nt = untreated; n.p. = not present category. For each column of each year, values followed by the same letter do not differ statistically according to Tukey's honest significant difference (HSD) test at p = 0.05.
